# Materia Medica or Pharmacology and Therapeutics

**Published:** 1856-01

**Authors:** 


*Meteria Medica or Pharmacology and Therapeutics.* By William Tully, M. D.

This is the title (rather long, if it could be curtailed) of a work, which, although it has reached it thirteenth number, is, nevertheless, new to us. We infer from the disconnected and disjointed portion contained in this number of its "Proem to Narcotics," that is a valuable work, but cannot say more, because we have not seen, as we said before, the other numbers. It is published monthly at Springfield, Mass., by Jefferson Church, M. D., at the rate of one dollar for every four numbers.

